# Supplementary material for: Rumen Microbiome Reveals the Differential Response of CO2 and CH4 Emissions of Yaks to Feeding Regimes on the Qinghai–Tibet Plateau
Source: Animals (Basel). 2022 Oct 30;12(21):2991. doi: 10.3390/ani12212991 (PMC9657323; doi:10.3390/ani12212991)
Supplement: Supplementary file 1 [file animals-12-02991-s001.zip › animals-1995926-supplementary.pdf]

Supplementary Material

Rumen Microbiome Reveals the Differential Response of CO_2 and CH_4 Emissions of Yaks to Feeding Regimes on the Qinghai–Tibet Plateau

Qian Zhang[1,2], Tongqing Guo[1,2], Xungang Wang[1], Xiaoling Zhang[1,2], Yuanyue Geng[1,2], Hongjin Liu[1], Tianwei Xu[1], Linyong Hu[1], Na Zhao[1], Shixiao Xu[1,*]

[1] Northwest Institute of Plateau Biology, Chinese Academy of Sciences, Xining 810008, China
[2] University of Chinese Academy of Sciences, Beijing 100049, China
* Correspondence: sxxu@nwipb.cas.cn

Table S1. Relative abundance of bacterial phylum levels of experimental groups.

Items	Groups			SEM	*P*-value
	YWG	YCG	YCF		
Relative abundance, %					
Firmicutes	0.4914	0.4745	0.4739	0.0183	0.8490
Bacteroidetes	0.4488	0.4826	0.4584	0.0169	0.8490
Proteobacteria	0.0276	0.0116	0.0387	0.0099	0.1580
Tenericutes	0.0044	0.0056	0.0057	0.0005	0.2030
TM7	0.0064	0.0029	0.0038	0.0007	0.0710
Synergistetes	0.0027	0.0062	0.0019	0.0011	0.7170
SR1	0.002	0.0039	0.0028	0.0005	0.4760
Verrucomicrobia	0.0045^{a}	0.0028^{ab}	0.0012^{b}	0.0005	0.0160
Actinobacteria	0.005	0.0016	0.0018	0.0007	0.0730
Spirochaetes	0.0006^{b}	0.0010^{ab}	0.0025^{a}	0.0003	0.0070
Others	0.0065	0.0074	0.0093	0.0010	0.1280

YWG: Warm-season grazing; YCG: Cold-season grazing; YCF: Cold-season indoor feeding; SEM: Standard error of the mean; Values in the same row with different letters are significantly different ($P < 0.05$).

Table S2. Relative abundance of fungal phylum levels of experimental groups.

Items	Groups			SEM [4]	*P*-value
	YWG	YCG	YCF		
Relative abundance, %					
Ascomycota	0.2889b	0.6774a	0.2270b	0.0563	0.0030
Basidiomycota	0.1594	0.1845	0.3148	0.0478	0.4020
Neocallimastigomycota	0.0021^{b}	0.0021^{b}	0.0779^{a}	0.0105	0.0030
Mucoromycota	0.0033^{ab}	0.0005^{b}	0.0288^{a}	0.0044	0.0010
Others	0.5456^{a}	0.1359^{b}	0.3432^{ab}	0.0601	0.0140

YWG: Warm-season grazing; YCG: Cold-season grazing; YCF: Cold-season indoor feeding; SEM: Standard error of the mean; Values in the same row with different letters are significantly different ($P < 0.05$).

Table S3. Relative abundance of archaeal phylum levels of experimental groups.

Items	Groups			SEM [4]	*P*-value
	YWG	YCG	YCF		
Relative abundance, %					
Euryarchaeota	0.9999[b]	0.9998[b]	1.0000[a]	0.00003	0.042

YWG: Warm-season grazing; YCG: Cold-season grazing; YCF: Cold-season indoor feeding; SEM: Standard error of the mean; Values in the same row with different letters are significantly different ($P < 0.05$).

Table S4. Functional genera of the rumen microbiome

	Family/Order	Genus	Functional description	References
Bacteria	Ruminococcaceae	*Bacteroides*<br>*Prevotella*<br>*Ruminococcus*	Polysaccharide degradation: bacterial groups capable of degrading cellulose, hemicellulose (xylan/xyloglucan) and pectin	[1,2]
Bacteria	Bacteroidaceae	*Bacteroides*	Carbohydrate degradation and Glycolysis in the rumen	[3]
Bacteria		*Butyrivibrio*<br>*Prevotella*<br>*Ruminococcus*	Polysaccharide-degrading genera	[4]
Archaea		*Methanobrevibacter*<br>*Methanobacterium*	Hydrogen-consuming methanogenic bacteria	[5]
Bacteria	Ruminococcaceae<br>Bacteroidaceae	*Prevotella*<br>*Ruminococcus Butyrivibrio*	Enabling the degradation of various hemicellulose and pectin polymers (xylan, mannan, arabinan and galactan)<br>Hemicellulose degradation	[3]
Bacteria	Bacteroidaceae<br>Prevotellaceae	*Prevotella*<br>*Fibrobacter*	Within microbes, often assist in the degradation of cellulose, hemicellulose and starch	[5]
Bacteria	Bacteroidaceae	*Butyrivibrio*	The vital saccharolytic role of Butyrivibrio in rumen carbohydrate degradation	[6]
Bacteria	Prevotellaceae<br>Ruminococcaceae<br>Bacteroidaceae	*Prevotella*<br>*Selenomonas*	Digesting multiple carbohydrate substrates	[7]
Archaea	Euryarcheota	*Methanobrevibacter*<br>*Methanobacterium*	Methanogenic archaea which produce CH_4	[8]
Bacteria		*Fibrobacter*	Degrading cellulose and hemi-celluloses	[9]

		Ruminococcus		
Bacteria		*Prevotella* *Selenomonas* *Butyrivibrio*	Degradind starch, hemi-celluloses	[5]
Fungi	Neocallimastigaceae	*Neocallimastix* *Orpinomyces*	Degrading plant cell wall carbohydrates	[10]
Archaea	Euryarcheota	*Methanobrevibacter* *Methanobacterium*	Exhibited high lignocellulose-degrading activity with the production of -CH_4 and acetate	[10]
Fungi Archaea	Neocallimastigaceae	*Neocallimastix* *Methanobrevibacter*	The importance of fungi as substrate and electron donors for methanogenesis	[11,12]
Bacteria	Prevotellaceae	*Fibrobacter*	Ruminal microorganisms cleave complex glycosidic bonds mainly through glycoside hydrolases	[5]
Archaea	Euryarcheota	*Methanobrevibacter*	Hydrogenotrophic methanogenesis pathways	[13-15]
Bacteria		*Fibrobacter* *Selenomonas*	Glycoside hydrolases	[5]
Bacteria Archaea		*Prevotella* *Methanobrevibacter*	Its was associated with the CH_4 yield	[16,17]
Bacteria		*Clostridiales* *Bacteroidales* *Selenomonadales*	Fermentative and bifurcating hydrogenases are highly expressed	[18]
Bacteria Archaea	Lachnospiraceae Ruminococcaceae	*Butyrivibrio Ruminococcus* *Methanobrevibacter Fibrobacter* *Prevotella* *Bacteroides*	Acetogens microorganisms degrade multiple substrates It was the assigned for methanogenesis	[19]
Bacteria Archaea		*Bacteroides* *Methanobrevibacter* *Ruminococcus Fibrobacter* *Prevotella*	Which mediate the process of electron confurcation during fermentative carbohydrate degradation leading to H_2 production	[20]
Bacteria		*Fibrobacter* *Ruminococcus*	Fibrolytic bacteria	[21]
Bacteria		*Selenomonas*	Supports hydrogenotrophic respiration and lower CH_4	[18]
Archaea		*Methanobrevibacter*	Its was associated with the CH_4 yield	[22]
Fungi	Neocallimastix	*Methanobrevibacter*	Its was associated with the	[23]

Archaea			CH_4 yield	
Bacteria		*Fibrobacter*	Contributes to the degradation offiber in the rumen	[24,25]
Bacteria		*Ruminococcus Fibrobacter Prevotella*	Played a pivotal role in polysaccharide degradation	[25,26]
Archaea Fungi		*Methanobrevibacter Orpinomyces*	Higher lignocellulose degrading and methane producing ability	[27]
Fungi		*Orpinomyces*	Capable of simultaneous saccharification and fermentation of the cellulosic and hemicellulosic fractions in multiple untreated grasses and crop residues examined	[28]
Bacteria		*Butyrivibrio*	In biomass degradation and conversion within the rumen.	[29]
Bacteria		*Fibrobacter Ruminococcus Prevotella*	The plant cell wall polysaccharide degradation	[30]
Archaea		*Methanobacterium*	Its was associated with the CH_4 yield	[31]
Bacteria		*Selenomonas Ruminococcus*	Its was associated with the CH_4 yield	[32]

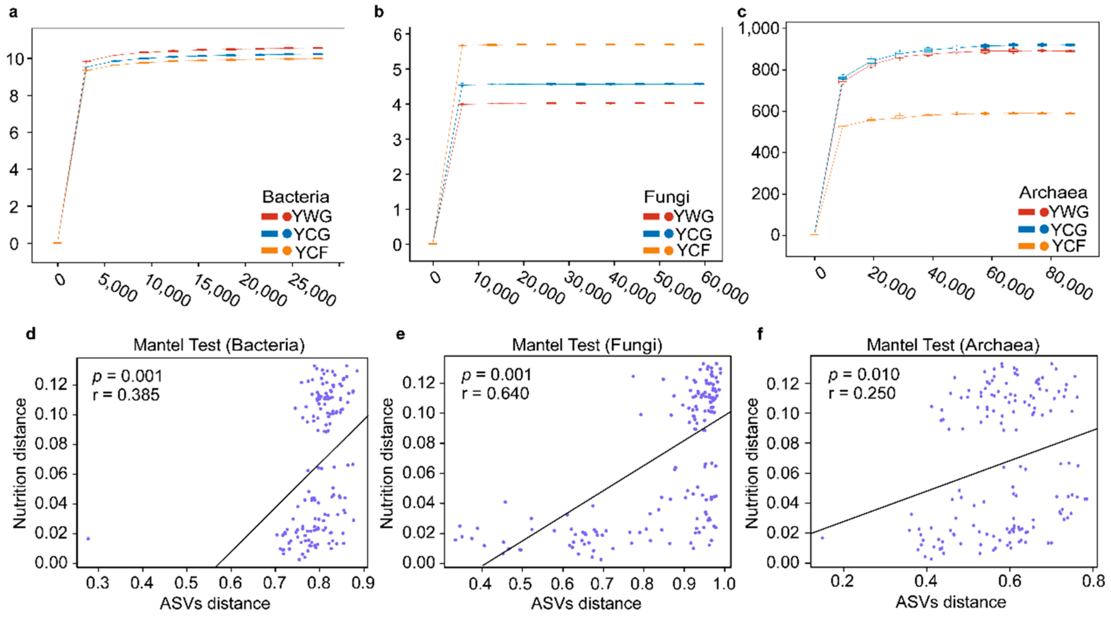

Figure S1. Rarefaction curves of the bacteria (a), fungi (b), archaea (c) gene reads based on ASVs. Mantel test revealed the correlation between rumen bacteria (d), fungi (e), archaea (f) and dietary nutrition (ASV level).

References

1. Ndeh, D.; Rogowski, A.; Cartmell, A.; Luis, A.S.; Baslé, A.; Gray, J.; Venditto, I.; Briggs, J.; Zhang, X.; Labourel, A.; et al. Complex pectin metabolism by gut bacteria reveals novel catalytic functions. *Nature* **2017**, *544*, 65-70, doi:10.1038/nature21725.
2. Henderson, G.; Cox, F.; Ganesh, S.; Jonker, A.; Young, W.; Abecia, L.; Angarita, E.; Aravena, P.; Nora Arenas, G.; Ariza, C.; et al. Rumen microbial community composition varies with diet and host, but a core microbiome is found across a wide geographical range. *Sci Rep* **2015**, *5*, 14567, doi:10.1038/srep14567.
3. Solden, L.M.; Naas, A.E.; Roux, S.; Daly, R.A.; Collins, W.B.; Nicora, C.D.; Purvine, S.O.; Hoyt, D.W.; Schückel, J.; Jørgensen, B.; et al. Interspecies cross-feeding orchestrates carbon degradation in the rumen ecosystem. *NAT. MICROBIOL* **2018**, *3*, 1274-1284, doi:10.1038/s41564-018-0225-4.
4. Seshadri, R.; Leahy, S.C.; Attwood, G.T.; Teh, K.H.; Lambie, S.C.; Cookson, A.L.; Eloe-Fadrosh, E.A.; Pavlopoulos, G.A.; Hadjithomas, M.; Varghese, N.J.; et al. Cultivation and sequencing of rumen microbiome members from the Hungate1000 Collection. *Nat. Biotechnol.* **2018**, *36*, 359-367, doi:10.1038/nbt.4110.
5. Stewart, R.D.; Auffret, M.D.; Warr, A.; Walker, A.W.; Roehe, R.; Watson, M. Compendium of 4,941 rumen metagenome-assembled genomes for rumen microbiome biology and enzyme discovery. *Nat. Biotechnol.* **2019**, *37*, 953-961, doi:10.1038/s41587-019-0202-3.
6. Xue, M.-Y.; Wu, J.-J.; Xie, Y.-Y.; Zhu, S.-L.; Zhong, Y.-F.; Liu, J.-X.; Sun, H.-Z. Investigation of fiber utilization in the rumen of dairy cows based on metagenome-assembled genomes and single-cell RNA sequencing. *Microbiome 10*, doi:10.1186/s40168-021-01211-w.
7. Janssen, P.H. Influence of hydrogen on rumen methane formation and fermentation balances through microbial growth kinetics and fermentation thermodynamics. *Anim. Feed Sci. Technol.* **2010**, *160*, 1-22, doi:10.1016/j.anifeedsci.2010.07.002.
8. Xie, F.; Jin, W.; Si, H.; Yuan, Y.; Tao, Y.; Liu, J.; Wang, X.; Yang, C.; Li, Q.; Yan, X.; et al. An integrated gene catalog and over 10,000 metagenome-assembled genome s from the gastrointestinal microbiome of ruminants. *Microbiome 9*, 137, doi:10.1186/s40168-021-01078-x.
9. Weimer, P.J. Why don't ruminal bacteria digest cellulose faster? *J Dairy Sci 79*, 1496-1502, doi:10.3168/jds.S0022-0302(96)76509-8.
10. Wei, Y.Q.; Yang, H.J.; Luan, Y.; Long, R.J.; Wu, Y.J.; Wang, Z.Y. Isolation, identification and fibrolytic characteristics of rumen fungi grown with indigenous methanogen from yaks (Bos grunniens) grazing on the Qinghai-Tibetan Plateau. *J. Appl. Microbiol.* **2016**, *120*, 571-587, doi:10.1111/jam.13035.
11. Cheng, Y.F.; Jin, W.; Mao, S.Y.; Zhu, W.-Y. Production of Citrate by Anaerobic Fungi in the Presence of Co-culture Methanogens as Revealed by 1H NMR Spectrometry. *Asian-Australas J Anim Sci* **2013**, *26*, 1416-1423, doi:10.5713/ajas.2013.13134.
12. Li, Y.; Meng, Z.; Xu, Y.; Shi, Q.; Ma, Y.; Aung, M.; Cheng, Y.; Zhu, W. Interactions between Anaerobic Fungi and Methanogens in the Rumen and Their Biotechnological Potential in Biogas Production from Lignocellulosic Materials. *Microorganisms* **2021**, *9*, doi:10.3390/microorganisms9010190.
13. Wallace, R.J.; Rooke, J.A.; McKain, N.; Duthie, C.-A.; Hyslop, J.J.; Ross, D.W.; Waterhouse,

A.; Watson, M.; Roehe, R. The rumen microbial metagenome associated with high methane production in cattle. *BMC Genomics* **2015**, *16*, 839, doi:10.1186/s12864-015-2032-0.

14. Cunha, C.S.; Marcondes, M.I.; Veloso, C.M.; Mantovani, H.C.; Pereira, L.G.R.; Tomich, T.R.; Dill-McFarland, K.A.; Suen, G. Compositional and structural dynamics of the ruminal microbiota in dairy heifers and its relationship to methane production. *J. Sci. Food Agric.* **2019**, *99*, 210-218, doi:10.1002/jsfa.9162.
15. Danielsson, R.; Dicksved, J.; Sun, L.; Gonda, H.; Müller, B.; Schnürer, A.; Bertilsson, J. Methane Production in Dairy Cows Correlates with Rumen Methanogenic and Bacterial Community Structure. *Front. Microbiol.* **2017**, *8*.
16. Liu, C.; Li, X.H.; Chen, Y.X.; Cheng, Z.H.; Duan, Q.H.; Meng, Q.H.; Tao, X.P.; Shang, B.; Dong, H.M. Age-Related Response of Rumen Microbiota to Mineral Salt and Effects of Their Interactions on Enteric Methane Emissions in Cattle. *Microb. Ecol.* **2017**, *73*, 590-601, doi:10.1007/s00248-016-0888-4.
17. Aguilar-Marin, S.B.; Betancur-Murillo, C.L.; Isaza, G.A.; Mesa, H.; Jovel, J. Lower methane emissions were associated with higher abundance of rumin al Prevotella in a cohort of Colombian buffalos. *BMC Microbiol. 20*, 364, doi:10.1186/s12866-020-02037-6.
18. Greening, C.; Geier, R.; Wang, C.; Woods, L.C.; Morales, S.E.; McDonald, M.J.; Rushton-Green, R.; Morgan, X.C.; Koike, S.; Leahy, S.C.; et al. Diverse hydrogen production and consumption pathways influence methane production in ruminants. *ISME J. 13*, 2617-2632, doi:10.1038/s41396-019-0464-2.
19. Pope, P.B.; Smith, W.; Denman, S.E.; Tringe, S.G.; Barry, K.; Hugenholtz, P.; McSweeney, C.S.; McHardy, A.C.; Morrison, M. Isolation of Succinivibrionaceae implicated in low methane emissions f rom Tammar wallabies. *Science 333*, 646-648, doi:10.1126/science.1205760.
20. Wang, S.; Huang, H.; Kahnt, J.; Thauer, R.K. A reversible electron-bifurcating ferredoxin- and NAD-dependent [FeFe] -hydrogenase (HydABC) in Moorella thermoacetica. *J. Bacteriol. 195*, 1267-1275, doi:10.1128/JB.02158-12.
21. Li, Q.S.; Wang, R.; Ma, Z.Y.; Zhang, X.M.; Jiao, J.Z.; Zhang, Z.G.; Ungerfeld, E.M.; Yi, K.L.; Zhang, B.Z.; Long, L.; et al. Dietary selection of metabolically distinct microorganisms drives hydr ogen metabolism in ruminants. *ISME J.*, doi:10.1038/s41396-022-01294-9.
22. Bauchop, T.; Mountfort, D.O. Cellulose Fermentation by a Rumen Anaerobic Fungus in Both the Absence and the Presence of Rumen Methanogens. *Appl. Environ. Microbiol. 42*, 1103-1110, doi:10.1128/aem.42.6.1103-1110.1981.
23. Li, Y.; Jin, W.; Cheng, Y.; Zhu, W. Effect of the Associated Methanogen Methanobrevibacter thaueri on the Dynamic Profile of End and Intermediate Metabolites of Anaerobic Fungu s Piromyces sp. F1. *Curr. Microbiol. 73*, 434-441, doi:10.1007/s00284-016-1078-9.
24. Comtet-Marre, S.; Parisot, N.; Lepercq, P.; Chaucheyras-Durand, F.; Mosoni, P.; Peyretaillade, E.; Bayat, A.R.; Shingfield, K.J.; Peyret, P.; Forano, E. Metatranscriptomics Reveals the Active Bacterial and Eukaryotic Fibrol ytic Communities in the Rumen of Dairy Cow Fed a Mixed Diet. *Front. Microbiol. 8*, doi:10.3389/fmicb.2017.00067.
25. Liang, J.; Zheng, W.; Zhang, H.; Zhang, P.; Cai, Y.; Wang, Q.; Zhou, Z.; Ding, Y. Transformation of bacterial community structure in rumen liquid anaero bic digestion of rice straw. *Environ. Pollut. 269*, 116130, doi:10.1016/j.envpol.2020.116130.
26. Ozbayram, E.G.; Kleinsteuber, S.; Nikolausz, M.; Ince, B.; Ince, O. Bioaugmentation of

anaerobic digesters treating lignocellulosic feedst ock by enriched microbial consortia. *Eng. Life Sci. 18*, 440-446, doi:10.1002/elsc.201700199.
27. Ma, Y.; Li, Y.; Li, Y.; Cheng, Y.; Zhu, W. The enrichment of anaerobic fungi and methanogens showed higher lignoc ellulose degrading and methane producing ability than that of bacteria and methanogens. *World J. Microbiol. Biotechnol. 36*, 125, doi:10.1007/s11274-020-02894-3.
28. Youssef, N.H.; Couger, M.B.; Struchtemeyer, C.G.; Liggenstoffer, A.S.; Prade, R.A.; Najar, F.Z.; Atiyeh, H.K.; Wilkins, M.R.; Elshahed, M.S. The Genome of the Anaerobic Fungus Orpinomyces sp. Strain C1A Reveals the Unique Evolutionary History of a Remarkable Plant Biomass Degrader. *Appl. Environ. Microbiol. 79*, 4620-4634, doi:10.1128/aem.00821-13.
29. Hagen, L.H.; Brooke, C.G.; Shaw, C.A.; Norbeck, A.D.; Piao, H.; Arntzen, M.Ø.; Olson, H.M.; Copeland, A.; Isern, N.; Shukla, A.; et al. Proteome specialization of anaerobic fungi during ruminal degradation of recalcitrant plant fiber. *ISME J 15*, 421-434, doi:10.1038/s41396-020-00769-x.
30. Dai, X.; Tian, Y.; Li, J.; Su, X.; Wang, X.; Zhao, S.; Liu, L.; Luo, Y.; Liu, D.; Zheng, H.; et al. Metatranscriptomic Analyses of Plant Cell Wall Polysaccharide Degradat ion by Microorganisms in the Cow Rumen. *Appl. Environ. Microbiol. 81*, 1375-1386, doi:10.1128/aem.03682-14.
31. Hirakata, Y.; Hatamoto, M.; Oshiki, M.; Watari, T.; Araki, N.; Yamaguchi, T. Food selectivity of anaerobic protists and direct evidence for methane production using carbon from prey bacteria by endosymbiotic methanoge n. *ISME J. 14*, 1873-1885, doi:10.1038/s41396-020-0660-0.
32. Kamke, J.; Kittelmann, S.; Soni, P.; Li, Y.; Tavendale, M.; Ganesh, S.; Janssen, P.H.; Shi, W.; Froula, J.; Rubin, E.M.; et al. Rumen metagenome and metatranscriptome analyses of low methane yield s heep reveals a Sharpea-enriched microbiome characterised by lactic aci d formation and utilisation. *Microbiome 4*, doi:10.1186/s40168-016-0201-2.
